# Cutoff value determines the performance of a semi-quantitative immunochemical faecal occult blood test in a colorectal cancer screening programme

**DOI:** 10.1038/sj.bjc.6605326

**Published:** 2009-09-15

**Authors:** L G M van Rossum, A F van Rijn, R J F Laheij, M G H van Oijen, P Fockens, J B M J Jansen, A L M Verbeek, E Dekker

**Affiliations:** 1 Department of Gastroenterology and Hepatology, Radboud University Nijmegen Medical Center, P.O.Box 9101, 6500 HB, Nijmegen, The Netherlands; 2 Department of Gastroenterology and Hepatology, Academic Medical Center, University of Amsterdam, Amsterdam, The Netherlands; 3 Department of Epidemiology and Biostatistics and MTA, Radboud University Nijmegen Medical Center, Nijmegen, The Netherlands

**Keywords:** colorectal cancer, faecal occult blood test, screening, epidemiology, colonoscopy

## Abstract

**Background:**

The cutoff of semi-quantitative immunochemical *faecal occult blood tests* (iFOBTs) influences colonoscopy referrals and detection rates. We studied the performance of an iFOBT (OC-Sensor) in colorectal cancer (CRC) screening at different cutoffs.

**Methods:**

Dutch screening participants, 50–75 years of age, with average CRC risk and an iFOBT value ⩾50 ng ml^−1^ were offered colonoscopy. The detection rate was the percentage of participants with CRC or advanced adenomas (⩾10 mm, ⩾20% villous, high-grade dysplasia). The *number needed to scope* (NNTScope) was the number of colonoscopies to be carried out to find one person with CRC or advanced adenomas.

**Results:**

iFOBT values ⩾50 ng ml^−1^ were detected in 526 of 6157 participants (8.5%) and 428 (81%) underwent colonoscopy. The detection rate for advanced lesions (28 CRC and 161 with advanced adenomas) was 3.1% (95% confidence interval: 2.6–3.5%) and the NNTScope was 2.3. At 75 ng ml^−1^, the detection rate was 2.7%, the NNTScope was 2.0 and the CRC miss rate compared with 50 ng ml^−1^ was <5% (*N*=1). At 100 ng ml^−1^, the detection rate was 2.4% and the NNTScope was <2. Compared with 50 ng ml^−1^, up to 200 ng ml^−1^ CRC miss rates remained at 16% (*N*=4).

**Conclusions:**

Cutoffs below the standard 100 ng ml^−1^ resulted in not only higher detection rates of advanced lesions but also more colonoscopies. With sufficient capacity, 75 ng ml^−1^ might be advised; if not, up to 200 ng ml^−1^ CRC miss rates are acceptable compared with the decrease in performed colonoscopies.

Compared with the guaiac-based FOBT (G-FOBT), one of the main advantages of some immunochemical faecal occult blood tests (iFOBTs) is that they allow haemoglobin quantification (Itoh *et al*, 1996; Castiglione *et al*, 2002; Guittet *et al*, 2007). The semi-quantitative nature of these tests permits adjustment of the cutoff value for the detection of colorectal cancer (CRC) in an effort to optimise screening programmes for specific populations and health-care practices. Changing the cutoff value can have considerable implications on the performance of the test in a screening population. In general, lowering the cutoff value will increase sensitivity, but consequently decrease specificity and vice versa. An increase in sensitivity means an increase in the detection of patients with colorectal cancer or advanced adenomas, but the consequential decrease in specificity results in more persons without relevant lesions undergoing a colonoscopy (false positives). Some studies in screening populations have been published on changing the cutoff value of iFOBTs. However, in these studies only a few selected cutoff values are presented, and the complete range of possible cutoff values is not addressed (Castiglione *et al*, 2002; Guittet *et al*, 2007; Grazzini *et al*, 2009). Furthermore, colonoscopy data, verifying the presence or absence of pathology, are usually presented for test results equal to or above the threshold that is recommended by the manufacturer. The two most frequently presented quantitative iFOBTs, the OC-Sensor (Eiken Chemical) and the Magstream 1000 (Fujirebio Diagnostics, Tokyo, Japan), were developed in Japan, where incidence rates for CRC are lower than those in Europe (Minami *et al*, 2006). Therefore, the cutoff value with optimal overall performance may be different in Europe compared with Japan. In a recent study including 1000 symptomatic and other high-risk patients in Israel, cutoff values below the recommended threshold of 100 ng ml^−1^ were evaluated (Levi *et al*, 2007). The authors concluded that the optimal cutoff value might be as low as 75 ng ml^−1^; they also noted that the test performance in average-risk patients in a screening population is unknown. Our aim was to evaluate the performance and efficiency of a semi-quantitative iFOBT in an average-risk screening population.

## Materials and methods

### Design and population

Details of study design and of most materials and methods relevant for this study are published elsewhere (Van Rossum *et al*, 2008). The study was primarily designed as a randomised controlled trial in an average-risk screening population between 50 and 75 years of age comparing a G-FOBT with an iFOBT. Here, we only describe data of the semi-quantitative iFOBT, OC-Sensor, to evaluate the performance of the iFOBT at different cutoff values in a population-based CRC screening. The name and address of invited subjects were randomly retrieved from municipal databases and the iFOBT was sent directly with the screening invitation. After 2 weeks, a single written reminder was sent. For detailed information regarding the randomisation and invitation procedure, we refer to the earlier publication (Van Rossum *et al*, 2008).

### iFOBT

In this study, the automated semi-quantitative OC-Sensor (Eiken Chemical Co., Tokyo, Japan) was used. Faecal samples, preserved in a plastic container in a liquid buffer, were processed using an OC-Micro instrument (Eiken Chemical Co.) (Levi *et al*, 2007). As threshold for positivity of the test, the manufacturer recommends a cutoff value of 100 ng ml^−1^, which has been applied in several studies (Castiglione *et al*, 2002; Grazzini *et al*, 2004; Sohn *et al*, 2005; Chiang *et al*, 2006; Fenocchi *et al*, 2006; Rubeca *et al*, 2006; Van Rossum *et al*, 2008). The literature and data provided by the manufacturer show that the test results of the OC-Sensor are reliable in the range from 50 to 2000 ng ml^−1^ (Vilkin *et al*, 2005), but in rare cases, the results measured can be much higher. In a previous publication, we compared the G-FOBT Hemoccult with the iFOBT OC-Sensor (Van Rossum *et al*, 2008). In that publication, for generalisability with previous studies, we presented data for the iFOBT with a fixed cutoff value of 100 ng ml^−1^. However, we invited all patients with an iFOBT result of ⩾50 ng ml^−1^ for colonoscopy, which data we use in this analysis. Below 50 ng ml^−1^, test results may become gradually more unreliable and, to our knowledge, no data on test reliability are available for cutoff levels below 50 ng ml^−1^, corresponding with ±10 *μ*g g^−1^ faeces (Levi *et al*, 2007).

### Colonoscopy

Colonoscopy was offered to all iFOBT-positive patients. All colonoscopies were carried out by experienced gastroenterologists using conscious sedation with midazolam and fentanyl. If the caecum could not be reached at the initial colonoscopy, the procedure was repeated using propofol anaesthesia, or a computerised tomography colonoscopy was carried out (followed by a second colonoscopy if necessary).

During colonoscopy, all polyps and colorectal cancer were removed if possible, and other lesions were biopsied if necessary. Advanced adenomas were defined as adenomas with a size of ⩾10 mm, adenomas with a villous component of ⩾20%, or adenomas with high-grade dysplasia. The villous component was judged by an experienced pathologist as being either completely villous or ⩾20% villous or <20% villous: if in doubt, a second opinion from another experienced GI pathologist was requested until consensus was reached. A similar consensus strategy was applied for high-grade dysplasia. Each CRC patient was staged according to the American Joint Committee on Cancer system (AJCC), also called the TNM system, which describes stages using Roman numerals I, II, III and IV (O’Connell *et al*, 2004). According to the Vienna Classification, carcinoma *in situ* or intramucosal carcinoma was not classified as CRC (Schlemper *et al*, 2000).

### Data analysis

In population-based colorectal cancer screening, only iFOBT-positive participants are followed-up with colonoscopy. Sensitivity is therefore not available in our study design. Specificity can be quite reliably estimated under the rare disease assumption as 1 minus the number of false positives relative to the total number of participants reduced by the number of true positives, disregarding the number of false FOBT-negative patients (negatives). (Brecht and Robra, 1987) We used detection rates and numbers needed to scope to evaluate the performance of iFOBT at different cutoff levels. Participants were defined as subjects who responded to the invitation by returning a used iFOBT. The detection rate, defined as the percentage of participants with colorectal cancer or ⩾1 advanced adenomas, was used to describe the yield of the test. Positivity rate was the percentage of positive participants and colonoscopy rate was the percentage of positive participants adherent to colonoscopy. Cancer miss rate was defined as the percentage of cancer patients missed relative to the number of cancer patients at the minimal cutoff value of 50 ng ml^−1^ of the iFOBT. The expense of the test was captured with the number needed to scope (NNTScope), representing the delicate ratio between true positives (all endoscoped iFOBT positives with CRC or ⩾1 advanced adenomas) and false positives (no or only minor neoplasia). The NNTScope as a reciprocal of the positive predictive value (PPV) (NNTScope= 1/PPV) is defined as the number of persons undergoing a colonoscopy to detect one person with CRC or ⩾1 advanced adenomas.

Detection rates and NNTScope were calculated and reported with 95% CI. In figures, detection rates and NNTScope are presented relative to the amount of haemoglobin (ng ml^−1^) on a continuous scale. Normal distribution of the iFOBT result was achieved by logarithmic transformation. The logarithmic mean difference in the amount of haemoglobin found in patients without cancer or advanced adenomas compared with that in patients with CRC or ⩾1 advanced adenoma was analysed with logistic regression analysis and reported with *P*-values. The influence of gender and age on logarithmic mean difference was evaluated with multivariable logistic regression analysis. Statistical analysis was carried out with SAS system for windows, software version 8.02 (SAS Institute Inc., Cary, NC, USA).

### Ethical approval and consent

The study was ethically reviewed and approved by the Dutch Health Council (2005/03WBO, The Hague, The Netherlands). All participants gave written informed consent for the iFOBT and, if positive, for colonoscopy.

## Results

Overall, 6157 (60%) of the 10 322 subjects invited to undergo the iFOBT underwent and returned the test. Women (63%) participated more often than men (56%) and persons <60 years of age a little less than persons ⩾60 years of age (59 *vs* 61%) (Table 1). A positive iFOBT, i.e., a test result ⩾50 ng ml^−1^, was found in 526 subjects, corresponding with a positivity rate of 8.5% (95% CI: 7.8–9.2) of participants. The positivity rate was 6.6% for women and 10.9% for men and, respectively, 6.4 and 10.8% for participants <60 and ⩾60 years of age. The age of one iFOBT positive female was unknown.

In 428 (81%) of these 526 patients, a colonoscopy was carried out, which constituted a colonoscopy rate (positive participants adherent to colonoscopy) of 7%. The caecum was reached in 402 patients (94%). In the 26 patients in whom the caecum could not be reached during the first colonoscopy, a successful second colonoscopy was carried out. In three of the stage III CRC patients, the second colonoscopy was not completed because of the obstructing tumour, instead a computerised tomography colonography was carried out.

### Per-polyp analysis

In total, 644 adenomas and 28 carcinomas were detected in 294 patients. Of the 266 patients without cancer, 161 patients had in total 250 advanced adenomas, of which 167 (67%) were ⩾1 cm, 66 (26%) had high-grade dysplasia, 19 (8%) were completely villous and 164 (66%) were considered to have ⩾20% villous aspects.

### Per patient analysis

CRC was detected in 28 (7%) of the 428 patients, and in 161 (38%) patients at least 1 advanced adenoma was detected (Table 2). Thus, 189 patients had either CRC or ⩾1 advanced adenoma, which amounts to 44% (95% CI: 40–49%) of the patients who underwent colonoscopy for a positive test. Of the 161 patients with ⩾1 advanced adenoma, 137 (85%) had one or more adenomas ⩾1 cm and 20 patients (6%) had advanced adenomas on the basis of villous aspects only.

### iFOBT performance

The iFOBT test results ranged from 0 to 4186 ng ml^−1^ haemoglobin. The mean amount of haemoglobin detected in iFOBT-positive subjects without CRC or advanced adenomas was 314 ng ml^−1^ (95% CI: 239–388) (Table 2), which was significantly lower than the mean amount of 785 ng ml^−1^ (95% CI: 563–1008) in patients with CRC and 523 ng ml^−1^ (95% CI: 420–627) in patients with ⩾1 advanced adenoma. The amount of haemoglobin in the faecal sample does not follow a normal distribution; the median is lower than the mean. The difference in the amount of haemoglobin in the sample of patients with CRC or ⩾1 advanced adenoma compared with patients without significant lesions is therefore less pronounced than the averages indicated in Table 2. By logarithmic transformation of the test value, a normal distribution for the (log) amount of haemoglobin was reached. Univariable logistic regression analysis showed quantity–response relations for the log-transformed test value and the likelihood of finding CRC (*P*<0.0001), stage I or II CRC (*P*<0.01), stage III or IV CRC (*P*<0.001) or advanced adenomas (*P*<0.001). In reality, only four patients with a stage II CRC were observed, and none with stage IIb and stage IV. Correction with logistic regression analysis for the possible confounders, age and gender, did not change any of the relations found, and statistical significance was robust for all groups.

**Figure 1:**
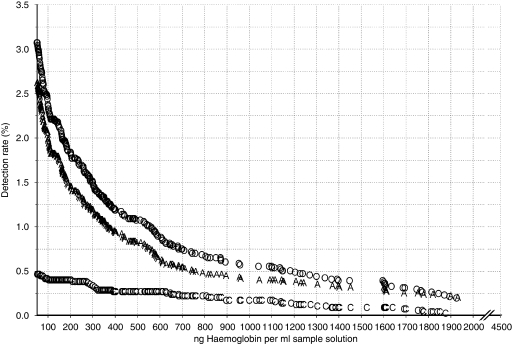
The detection rate (%) for colorectal cancer and advanced adenomas for the operative range of the immunochemical faecal occult blood test (iFOBT) (50–2000 ng ml^−1^). Overall detection rate of colorectal cancer and advanced adenomas (O), subgroup detection rates of colorectal cancer (C) and advanced adenomas (A) (adenomas ⩾10 mm, adenomas with high-grade dysplasia or adenomas with a villous component ⩾20%).

**Figure 2:**
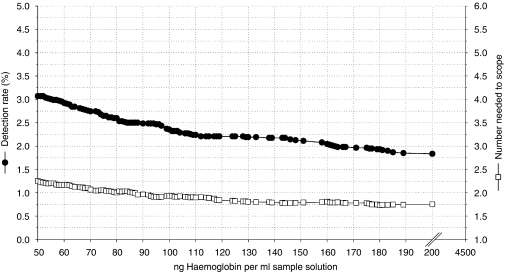
The overall detection rate and number needed to scope for cancer and ⩾1 advanced adenomas focused on the range between 50 and 200 ng ml^−1^. Left axis: detection rate for colorectal cancer and advanced adenomas (-•-) Right axis: number needed to scope for colorectal cancer and advanced adenomas (-□-) (by definition ⩾1.0).

Although the results ranged from 0 to 4186 ng ml^−1^, in Figure 1, the detection rates for CRC and advanced adenomas are presented for a maximum of 2000 ng ml^−1^. Besides the overall detection rates for CRC and advanced adenomas, separate detection rates for CRC and advanced adenomas are presented as well. At a cutoff value of 50 ng ml^−1^, the overall detection rate for CRC and advanced adenomas was 3.1% (95% CI: 2.6–3.5), and the NNTScope was 2.3 (95% CI: 2.2–2.3) (Figure 2 and Table 3). At the cutoff value >75 ng ml^−1^, the detection rate was 2.7% and the NNTScope was 2, that is, in every second colonoscopy, a patient with CRC or at least one advanced adenoma was detected. At this level one patient with stage I cancer was excluded on comparing with the cutoff value of 50 ng ml^−1^. At a cutoff value of 100 ng ml^−1^, the overall detection rate for CRC and advanced adenomas was 2.4% (95% CI: 2.0–2.7), and the NNTScope was 1.9 (95% CI: 1.9–2.0). At a cutoff value of 200 ng ml^−1^, the colonoscopy rate decreased by >50% compared with 50 ng ml^−1^, that is, 50% less colonoscopies had to be carried out. At this level, the detection rate for CRC and advanced adenomas was 1.8% (95% CI: 1.5–2.2) and the NNTScope was 1.8 (95% CI: 1.7–1.8). In the range from 100–200 ng ml^−1^, the cancer miss rate compared with the cutoff value ⩾50 ng ml^−1^ was stable at 14% (*N*=4).

According to Table 3, a decrease in the cutoff value from 100 ng ml^−1^ to 50 ng ml^−1^ resulted in increasing detection rates of advanced lesions from 2.4 to 3.1%, but at the cost of more colonoscopies that needed to be carried out, from 4.5 to 7%, and higher rates of colonoscopies without significant lesions. Increasing the cutoff value from 100 to 200 ng ml^−1^ decreases the detection rate for advanced adenomas substantially from 2.4 to 1.8%, not affecting the detection rate for cancer. The colonoscopy rate is reduced to 50% by using a cutoff value of 200 ng ml^−1^ compared with 50 ng ml^−1^ (3.2 *vs* 7%). The cancer miss rate at 200 ng ml^−1^ compared with 50 ng ml^−1^ is 14%.

**Figure 3:**
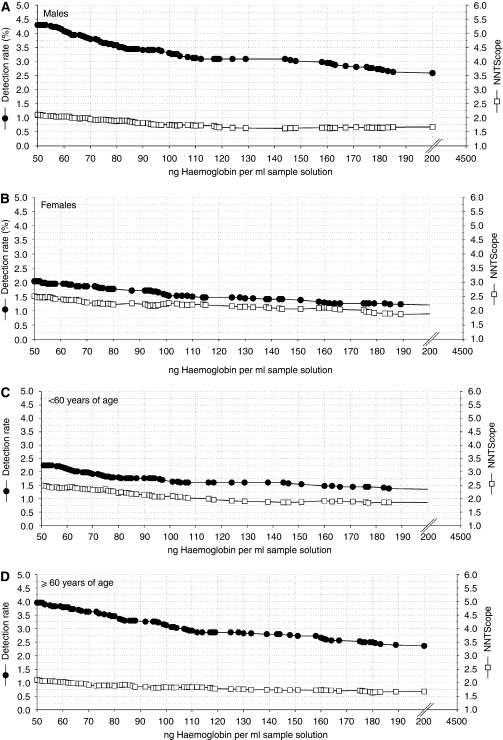
The detection rate (%) and number needed to scope for colorectal cancer and ⩾1 advanced adenomas focused on the range between 50 and 200 ng ml^−1^ for males (**A**) (*N*=257), for females (**B**) (*N*=171), patients <60 years of age (**C**) (*N*=252) and for patients ⩾60 years of age (**D**) (*N*=175). Left axis: detection rate for colorectal cancer and advanced adenomas (-•-). Right axis: number needed to scope (NNTScope) for colorectal cancer and advanced adenomas (-□-) (by definition ⩾1.0).

In Figure 3A–D, the relation between detection rates and NNTScope for subjects <60 years and ⩾60 years, and for men and women, is presented.

## Discussion

We evaluated the performance and efficiency of a semi-quantitative iFOBT, the OC-Sensor, in an average-risk screening population. We presented detection rates and numbers needed to scope over the complete effective range of the test, and we have shown that, below the threshold of 100 ng ml^−1^ recommended by the manufacturer of the test, even at the lowest functional value of 50 ng ml^−1^ tested, the performance could be considered acceptable. At the 50 ng ml^−1^ level, both cancer and advanced adenomas were detected at the cost of significantly more colonoscopies. At higher cutoff levels than the standard 100 ng ml^−1^, significantly less colonoscopies have to be carried out; moreover, compared with 100 ng ml^−1^, the cancer miss rate was nil but the miss rate of advanced adenomas was substantial.

In a previous publication, we demonstrated that the performance of the G-FOBT, Hemoccult-II, is lower than the performance of the iFOBT, OC-Sensor, at the standard cutoff value of 100 ng ml^−1^ (Van Rossum *et al*, 2008). The PPV for CRC and advanced adenomas of the G-FOBT was 55.3%, which was reached with the iFOBT at a cutoff of 130 ng ml^−1^. At this cutoff, the NNTScope of iFOBT was equal to that of G-FOBT, but the detection rate for CRC was more than 2.5 times higher for iFOBT compared with that for G-FOBT, and for advanced adenomas, this was almost twice as high.

The quantitative aspect of the iFOBT allows to adjust the cutoff value to a screening programme and can be based on aspects such as the intended detection rate, population-related factors (e.g., prevalence of CRC, participation rates) and political issues such as colonoscopy capacity. The cutoff value with the most optimal performance of the iFOBT may differ in various populations and may change over time, because the performance is dependent on the prevalence of CRC and advanced adenomas. Therefore, the data from this study should not be extrapolated to other populations, without considering these variables. However, we do think extrapolation of results will be quite appropriate for most European countries, as the prevalence of CRC and the capacity of adequate health care are comparable (Regula *et al*, 2006; Segnan *et al*, 2007).

With regard to colonoscopy capacity, the colonoscopy rate of 7% at the lowest cutoff value of 50 ng ml^−1^ could be considered acceptable if compared with that of countries with primary colonoscopy screening, Moreover, in case of a colonoscopy because of a positive iFOBT, the positive predictive value for advanced lesions will be substantially higher than in persons undergoing primary colonoscopy screening.

Previous studies published regarding semi-quantitative iFOBTs in screening populations did not address the true continuous nature of these tests (Castiglione *et al*, 2002; Guittet *et al*, 2007). Furthermore, these studies did not present colonoscopy results below the threshold recommended by the manufacturer. In a recent study on 1000 symptomatic and other high-risk patients in Israel with the same OC-Sensor test from Eiken Chemical, cutoff values below the recommended threshold were evaluated (Levi *et al*, 2007). The results of this study are comparable with our results, and the authors suggested that a cutoff level of 75 ng ml^−1^ would probably achieve optimal results, acknowledging the fact that population-based screening data are needed for confirmation.

A limitation of our study was that we only have data for one sample of iFOBT. In the previously mentioned study, 1000 symptomatic and other high-risk patients in Israel were asked to undergo three separate sample tests (Levi *et al*, 2007). The authors observed an increased sensitivity for more than one sample, although the difference between two and three samples was not significant and the specificity decreased when more samples were used. It is conceivable that a screening strategy with two samples results in a different optimal cutoff value when compared with one sample screening. However, an increase in sensitivity and therefore detection rate by two tests could be matched by a decrease in participation rate, resulting in a decrease in detection rate according to an *intention-to-screen analysis*.

It is unavoidable in population-based screening studies with a two tier model (that is, colonoscopy only under the condition of a positive pretest) to not offer a colonoscopy to iFOBT negatives; therefore, sensitivity of the iFOBT could not be calculated in this study. However, the sensitivity of the iFOBT could possibly be estimated for the purposes of cost-effectiveness studies, provided extensive sensitivity analyses are carried out for incidence and prevalence, because incidence and prevalence of colorectal cancer seem quite similar in most European countries (Regula *et al*, 2006; Ferlay *et al*, 2007; Segnan *et al*, 2007; Hundt *et al*, 2009).

Of the participants with a positive iFOBT, 19% did not adhere to the colonoscopy. Similar percentages have been observed in other population-based screening studies (Faivre *et al*, 2004). While designing our study (using municipal databases), we were unable to carry out a pre-selection according to eligibility. Therefore, this percentage consists of those individuals who refused to undergo colonoscopy, and also individuals who were excluded for colonoscopy because of, e.g., severe co-morbidity or recent colonoscopy. As we conducted an implementation study, we did not exclude these patients from the 19% who were not adherent to colonoscopy.

The villous component for advanced adenoma status can be difficult to evaluate, especially if the level is on the predefined limit of 20%. However, the percentage of patients with a colonoscopy with only a villous component as the criterion for advanced adenoma status was 6% of all patients. Of all patients with advanced adenomas, 85% had at least an adenoma ⩾10 mm in size. Even if all 20 patients with only a villous component had been excluded from the group with advanced adenomas, our conclusions would have remained identical.

Advanced adenomas and CRC were found more often in men than in women, and advanced adenomas and CRC were also more often detected in older persons. This is in line with other studies (Manus *et al*, 1997; Betes *et al*, 2003; Sedjo *et al*, 2007). Thus, the diagnostic yield increases with age and male gender. We showed that for gender and different age groups, other optimal cutoff values might be valid, but with the present data, we were unable to verify the validity of any specific recommendations. It might be interesting to investigate the possibility, as well as the acceptability, of age- and gender-specific cutoff values.

*In conclusion*: This study presents evidence that, below the standard threshold of 100 ng ml^−1^, which is recommended by the manufacturer of the iFOBT OC-Sensor, acceptable performance could be achieved even at the lowest functional value of 50 ng ml^−1^. Substantially higher detection rates for colorectal cancer and for advanced adenomas correspond with increasing numbers needed to be scoped. However, the positivity rate and the resulting number of colonoscopies to be carried out are relatively high compared with higher cutoff values (100–200 ng ml^−1^). We propose that a cutoff value below 100 ng ml^−1^ would be feasible and acceptable in screening programmes in many Western European countries, assuming resources and colonoscopy capacity are sufficient. However, when resources and colonoscopy capacity are not sufficient, cutoff values above 100 ng ml^−1^ (up to 200 ng ml^−1^) will result in a relatively limited miss rate for colorectal cancer and a lower but still quite high detection rate of advanced adenomas at the expense of a decrease in the total number of colonoscopies to be carried out. Policy makers will determine the optimal cutoff value on the basis of a largely arbitrary balance between the acceptability of missing cancer and the possibility and acceptability of assigning essential resources. In the Netherlands, there is indication that a cutoff of 75 ng ml^−1^ is considered for implementation in national screening.

## Figures and Tables

**Table 1:**
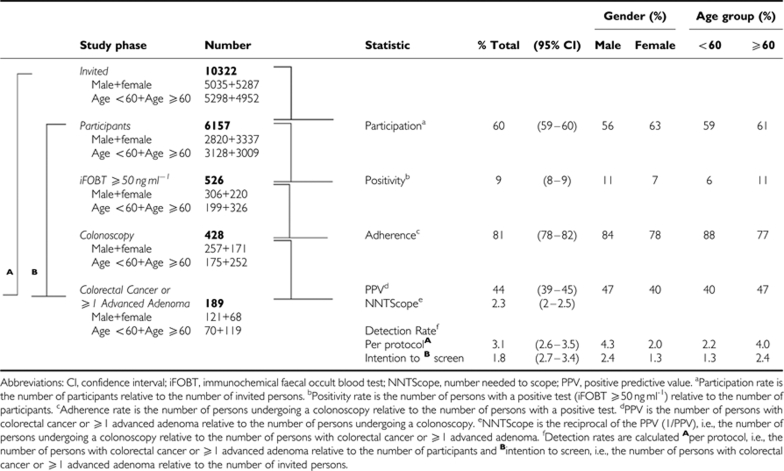
Basic numbers and descriptive statistics of the study population invited to perform an iFOBT in population based colorectal cancer screening

**Table 2:** Characteristics of lesions found at colonoscopy and iFOBT result (ng ml^−1^)

				**Range**		**Percentile**		
**iFOBT positives (⩾50 ng ml^−1^)**	***N***	**(%)**	**Mean (95% CI)**	**Min**	**Max**	**25th**	**Median**	**75th**
Without colonoscopy	98	(19)	455 (326–583)	52	2916	76	144	497
With colonoscopy	428	(81)	434 (377–492)	50	4168	80	175	482
Neither cancer nor adenomas^a^	134	(31)	314 (239–388)	50	2818	76	125	347
Non-advanced Adenomas^b^	105	(25)	356 (232–481)	50	4168	69	109	326
Advanced Adenomas^c^	161	(38)	523 (420–627)	52	3322	100	251	582
Cancer^d^	28	(7)	785 (563–1008)	59	1871	283	662	1226
Stage I or II cancer	19	(68)	652 (400–904)	59	1871	280	384	1099
Stage III or IV cancer	9	(32)	1066 (662–1470)	202	1845	622	1182	1635

Abbreviation: iFOBT, immunochemical faecal occult blood test.

a Neither cancer nor adenomas: colonoscopies without any lesions or only hyperplastic polyps, serrated adenomas or unclassified polyps.

b Non advanced adenomas: colonoscopy with adenomas but without advanced adenomas and cancer.

c Advanced adenomas: colonoscopies without cancer but with adenomas ⩾10 mm in size, high grade dysplasia or a villous component ⩾20%.

d Staging according to TNM classification: there were only 4 stage II (all stage IIa, none stage IIb) and no stage IV patients. Therefore, stages I and II, and III and IV were combined.

**Table 3:** The performance characteristics of the iFOBT, OC-Sensor, at different cutoff levels

	**Cutoff values (ng ml^−1^)**							
	**50**	**75**	**100**	**125**	**150**	**175**	**200**	**225**
*Positives adherent to colonoscopy*^a^ *(N)*	428	336	280	248	234	215	198	187
*Colonoscopy rate*^b^ *(%)*	7.0%	5.5%	4.5%	4.0%	3.8%	3.5%	3.2%	3.0%
*Number of lesions (n)*								
Colorectal cancer	28	27	24	24	24	24	24	23
CRC+advanced adenomas	189	163	145	136	131	121	113	109
								
*Detection rate*^c^ *(%)*								
Colorectal cancer	0.45%	0.44%	0.39%	0.39%	0.39%	0.39%	0.39%	0.37%
Confidence interval (95% CI)	0.3–0.6%	0.3–0.6%	0.2–0.6%	0.2–0.6%	0.2–0.6%	0.2–0.6%	0.2–0.6%	0.2–0.5%
CRC+advanced adenomas	3.1%	2.6%	2.4%	2.2%	2.1%	2.0%	1.8%	1.8%
Confidence interval (95% CI)	2.6–3.5%	2.3–3.1%	2–2.7%	1.8–2.6%	1.8–2.5%	1.6–2.3%	1.5–2.2%	1.4–2.1%
								
*Number Needed To Scope*^d^ *(N/n)*								
Colorectal cancer	15.3	12.4	11.7	10.3	9.8	9.0	8.3	8.1
Confidence interval (95% CI)	11.3–23.8	9.1–19.5	8.4–18.9	7.5–16.7	7.1–15.7	6.5–14.4	6–13.2	5.9–13.2
CRC+advanced adenomas	2.3	2.1	1.9	1.8	1.8	1.8	1.8	1.7
Confidence interval (95% CI)	2.6–2.5	1.9–2.3	1.7–2.2	1.6–2.1	1.6–2	1.6–2	1.6–2	1.5–2
								
*Specificity* e								
CRC+advanced adenomas	96.0%	97.1%	97.8%	98.1	98.3	98.4	98.6	98.7
Confidence interval (95% CI)	95.5–96.5%	96.7–97.5%	97.4–98.1%	97.8–98.5%	98.0–98.6%	98.1–98.8%	98.3–98.9%	98.4–99.0%
*CRC miss rate*^f^ *(%)*	N.A.	3.6%	14.3%	14.3%	14.3%	14.3%	14.3%	17.9%
Confidence interval (95% CI)	N.A.	−3.3–10.4%	1.3–27.2%	1.3–27.2%	1.3–27.2%	1.3–27.2%	1.3–27.2%	3.7–32%

Abbreviations: CI, confidence interval; CRC, colorectal cancer; iFOBT, immunochemical faecal occult blood test.

a Postives adherent to colonoscopy=patients with a positive iFOBT who underwent a colonoscopy.

b Colonoscopy rate=percentage of participants with a positive iFOBT who underwent a colonoscopy.

c Detection rate=percentage of participants with lesions of reference.

d Number Needed To Scope=the number of patients to find one extra patient with lesions of reference.

e Specificity was calculated under the rare disease assumption (Brecht and Robra, 1987).

f CRC miss rate=the percentage of the colorectal cancer patients at that cutoff relative to the colorectal cancer patients at the minimal 50 ng ml^−1^ cutoff.
